# Cellular dynamics following CAR T cell therapy are associated with response and toxicity in relapsed/refractory myeloma

**DOI:** 10.1038/s41375-023-02129-y

**Published:** 2024-01-06

**Authors:** Luise Fischer, Nora Grieb, Patrick Born, Ronald Weiss, Sabine Seiffert, Andreas Boldt, Stephan Fricke, Paul Franz, Simone Heyn, Anne Sophie Kubasch, Ronny Baber, Heike Weidner, Song Yau Wang, Enrica Bach, Sandra Hoffmann, Jule Ussmann, Janine Kirchberg, Saskia Hell, Sebastian Schwind, Klaus H. Metzeler, Marco Herling, Madlen Jentzsch, Georg-Nikolaus Franke, Ulrich Sack, Kristin Reiche, Ulrike Köhl, Uwe Platzbecker, Vladan Vucinic, Maximilian Merz

**Affiliations:** 1 Department of Hematology, Hemostaseology, Cellular Therapy and Infectiology, University Hospital of Leipzig, Leipzig, Germany; 2 Innovation Center Computer Assisted Surgery (ICCAS), Leipzig, Germany; 3 Institute for Clinical Immunology, University Hospital of Leipzig, Leipzig, Germany; 4 Fraunhofer Institute for Cell Therapy and Immunology IZI, Leipzig, Germany; 5 Institute for Laboratory Medicine Clinical Chemistry and Molecular Diagnostics, University Hospital Leipzig, Leipzig, Germany; 6 Leipzig Medical Biobank, University Leipzig, Leipzig, Germany; 7 Bone Lab Dresden, University Hospital Dresden, Dresden, Germany

**Keywords:** Myeloma, Cancer immunotherapy

## Abstract

B-cell maturation antigen (BCMA)-targeting chimeric antigen receptor (CAR) T cells revolutionized the treatment of relapsed/refractory multiple myeloma (RRMM). However, data on cellular (CAR) T cell dynamics and the association with response, resistance or the occurrence of cytokine release syndrome (CRS) are limited. Therefore, we performed a comprehensive flow cytometry analysis of 27 RRMM patients treated with Idecabtagene vicleucel (Ide-cel) to assess the expansion capacity, persistence and effects on bystander cells of BCMA-targeting CAR T cells. Additionally, we addressed side effects, like cytokine release syndrome (CRS) and cytopenia. Our results show that in vivo expansion of CD8^+^ CAR T cells is correlated to response, however persistence is not essential for durable remission in RRMM patients. In addition, our data provide evidence, that an increased fraction of CD8^+^ T cells at day of leukapheresis in combination with successful lymphodepletion positively influence the outcome. We show that patients at risk for higher-grade CRS can be identified already prior to lymphodepletion. Our extensive characterization contributes to a better understanding of the dynamics and effects of BCMA-targeting CAR T cells, in order to predict the response of individual patients as well as side effects, which can be counteracted at an early stage or even prevented.

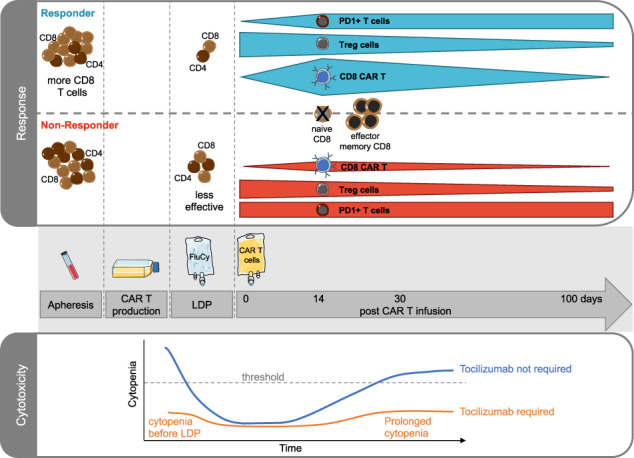

## Introduction

Chimeric antigen receptor (CAR) T cell therapy has revolutionized the treatment landscape for hematological malignancies, offering a new therapeutic avenue for patients with relapsed or refractory disease. Long-term follow-up data from CD19-CAR T cells indicate that they offer curative potential for a subset of patients, especially those with deep initial responses and lower baseline tumor volumes. Importantly, studies have shown that CD19-CAR T cells can persist for years, suggesting their contribution to sustained remissions and potential cure [1, 2]. Furthermore, positive effects on the non-CAR immune cell activation have been described in the ZUMA-1 trial [3]. While the efficacy of CD19-CAR T cells is well-documented [1, 4–8], data on B-cell Maturation Antigen (BCMA)-CAR T cells in multiple myeloma (MM) are still emerging. BCMA is an attractive target for CAR T cell therapy in MM due to its high and selective expression on malignant plasma cells. Early clinical trials have shown promising results, with high response rates, leading to FDA and EMA approval of Idecabtagene vicleucel (Ide-cel) and Ciltacabtagene autoleucel (Cilta-cel) for the treatment of triple-class exposed relapsed/refractory MM (RRMM) [9, 10]. The durability of these responses as well as long-term persistence of BCMA-CAR T cells are still not fully understood and effects on bystander T cells have not been investigated. Furthermore, the impact on side effects like cytokine release syndrome (CRS) and prolonged cytopenia needs to be studied.

In this study, we provide a comprehensive longitudinal analysis of CAR T and T cells after BCMA-CAR T cell therapy in RRMM. By leveraging advanced flow cytometry, we track the persistence of CAR T and T cells at multiple time points post-infusion. Our findings shed light on the unique features of BCMA-CAR T cell therapy and provide valuable insights for the optimization of CAR T cell therapies in RRMM.

## Patients and methods

### Sample collection and treatment procedure

This study was conducted in RRMM patients who underwent treatment with the commercial CAR T cell Ide-cel after three lines of therapy, including a proteasome inhibitor, immunomodulatory drug and anti-CD38 antibody. The treatment was administered at the University Hospital in Leipzig, Germany, in an in-patient setting. The treatment protocol included lymphodepletion (LDP) with cyclophosphamide and fludarabine in accordance with the KarMMa study. Data and sample collection were approved by the local ethics committee (361/22-ek and LMB-UCCL-2022_06) and were conducted in line with the Declaration of Helsinki. All patients gave written informed consent.

Peripheral blood (PB) samples were collected: on the day of leukapheresis, the day of CAR T cell infusion (day 0), and on days 7, 14, 30, and 100 post-treatment. Patient responses were monitored in our outpatient clinic and evaluated based on the International Myeloma Working Group (IMWG) recommendations. Serum samples were analyzed by ELISA to assess plasma levels of soluble BCMA (sBCMA).

### Immunophenotyping

PB of MM patients taken at the above-mentioned time points were analyzed by flow cytometry. The protocol for immunophenotyping was published recently [11]. Briefly, for analysis of the immune status including CAR detection, a BCMA-CAR Detection Reagent (Miltenyi Biotec, Bergisch Gladbach, Germany) in combination with the following antibodies was used: CD4, CD45, CD8, CD19, CD38, CD3, CD16, CD56, PD-1, HLA-DR and CD14 (all from Becton Dickinson [BD], Heidelberg, Germany). To analyze regulatory T (Treg) cells and T cell differentiation the antibody panels as described in Boldt et al. [12] were used.

Cells were analyzed using a FACSLyric flow cytometer and data analysis was performed using the FACSuite software (BD).

### Cytotoxicity assay

To assess functionality of CAR T cells, PB mononuclear cells (PBMCs) were isolated at days 0 and 7 using Pancoll (PAN-Biotech GmbH, Aidenbach, Germany) density centrifugation. PBMCs were cultured on CD3 antibody (Invitrogen/Thermo Fisher Scientific, Waltham, MA, USA) coated culture plates in X-Vivo medium (Lonza, Basel, Switzerland) supplemented with 2% AB-Serum (Sigma Aldrich/Merck KGaA, Darmstadt, Germany) (culture medium) as well as 200 U/ml IL-2 (Peprotech/Thermo Fisher Scientific) and stimulated with CD28 antibody (Invitrogen). These effector cells were cultured for up to 3 weeks to induce T cell proliferation.

The target cell line U266 maintained in RPMI1640 medium, was labeled with 1 µM VPD450 (BD) and incubated for 15 min at 37 °C. Afterwards, cells were washed with PBS and 30,000 target cells were co-cultured with 200,000 effector cells in culture medium in 96-well U-bottom plates for 24 h at 37 °C. 5000 flow cytometry count beads (Invitrogen) were added to quantify the number of cells. Target cells without the addition of effector cells were used as controls.

For analysis, cells were washed with PBS and stained with Fixable Viability Dye FVD eFluor® 780 (Thermo Fisher Scientific) for 15 min at 4 °C, and then washed with PBS + 2% FCS. They were subsequently incubated with 2.5 µl CD3 FITC antibody (Biolegend, San Diego, CA, USA) in 50 µl PBS + 2% FCS for 10 min at 4 °C. After washing, cells were fixed with 1% formaldehyde and analyzed using FACSCanto II flow cytometer (BD). Data analysis was performed using FlowJo 10.8.1 (FlowJo/BD).

### Statistical analyses

Statistical analysis was performed using R (version 4.3.1) and statistical tests were used as indicated in the respective figure legends. For survival analysis the R packages *survival* and *survminer* were used. Additionally, *ggplot2* and *ggpubr* were applied to generate the figures and significance levels. Statistical significance was evaluated with a threshold of *p* < 0.05.

## Results

### Patients

**Fig. 1:**
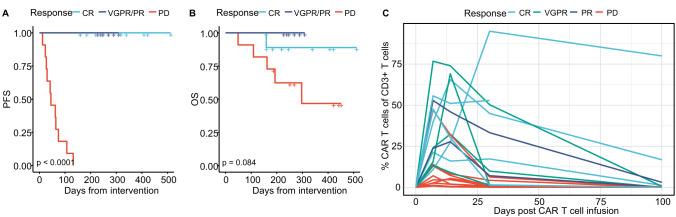
Response-related outcome and CAR T cell dynamics. **A**, **B** Progression-free survival (PFS) and overall survival (OS) curves of Ide-cel-treated RRMM patients classified according to their response into patients with complete response (CR, *n* = 10), with very good partial and partial remission (VGPR/PR, *n* = 6) as well as patients with progressive disease (PD, *n* = 11). **C** Percentages of CAR T cells within CD3^+^ T cell compartment in PB after CAR T cell infusion (day 0). For *p*-value calculation the log-rank test was used in (**A**) and (**B**).

We analyzed 27 heavily pretreated RRMM patients (median number of prior therapy lines: *n* = 7, range: 3–13) treated with Ide-cel. Response evaluation 100 days after CAR T cell infusion categorized patients into complete remission (CR, *n* = 10), very good partial and partial remission (VGPR/PR, *n* = 6), and progressive disease (PD, *n* = 11). Analysis of progression-free survival (PSF) revealed that while PD patients progressed already within the initial weeks after intervention, all CR and interestingly also all VGPR/PR patients remained without progression until day of last contact (Fig. 1A, median follow-up = 178 days). Similar trends were observed for overall survival (OS, Fig. 1B), with PD being the primary cause of death, except for one patient, who died from infection.

Assessment of CAR T cell dynamics post-infusion revealed, that the peak of CD3^+^ CAR T cells was detected two weeks after infusion and declined in PB within the following weeks with only a few exceptions (Fig. 1C). Notably, most patients had no detectable CAR T cells 100 days after infusion, regardless of remission. Detailed response characterization revealed a similar longitudinal CAR T cell development between CR, VGPR, and PR patients. For this reason and in combination with the PFS and OS results, patients were grouped according to their response into patients with PD (*n* = 11) and with response (PR or better, non-PD: *n* = 16) for all further analyses.

### Impact of patient characteristics on treatment with Ide-cel

Patient characteristics are summarized in Table 1. Several factors were associated with adverse outcome in our cohort: while the age at primary diagnosis was not significantly different, PD patients were younger at the time of CAR T cell infusion compared to non-PD patients. This was accompanied by a significantly shorter time between primary diagnosis and CAR T cell treatment (PD: 1168 days, non-PD: 2797 days, *p* = 0.001). In line with this observation, patients with CR or VGPR assessment prior to CAR T cell infusion showed sustained response after Ide-cel treatment and half of the patients with a suboptimal disease containment (PR or worse) did not respond to CAR T cell treatment. Expression of lambda light chain was furthermore associated with dismal outcome in our cohort (*p* < 0.0001). Assessment of sBCMA serum levels revealed significantly higher values in PD patient compared to responders s at all three different time points (apheresis: *p* < 0,01; day 30: *p* < 0.001 and day 100: *p* < 0.01, Supplementary Fig. 1). While there were no significant differences in sBCMA levels in PD patients during the observation period, sBCMA levels decreased significantly on day 30 and day 100 compared to serum collected at apheresis (*p* < 0.0001, respectively, Supplementary Fig. 2). No loss of BCMA expression was observed in both groups.

**Table 1:** Patient characteristics.

Feature	Stratification	non-PD (*N* = 16)	PD (*N* = 11)	*p*-value
Sex (NA = 0)	Female	9 (56.2%)	2 (18.2%)	*p* = 0.05
	Male	7 (43.8%)	9 (81.8%)	
ECOG (NA = 0)	0	2 (12.5%)	1 (9.1%)	*p* = 0.28
	1	11 (68.8%)	10 (90.9%)	
	2	3 (18.8%)	0 (0%)	
*t*(4;14) (NA = 2)	Yes	3 (20%)	2 (20%)	*p* = 1
	No	12 (80%)	8 (80%)	
*t*(14;16) (NA = 2)	Yes	0 (0%)	0 (0%)	*p* = NaN
	No	15 (100%)	10 (100%)	
del(17p) (NA = 2)	Yes	2 (13.3%)	2 (20%)	*p* = 0.66
	No	13 (86.7%)	8 (80%)	
ampl(1q) (NA = 2)	Yes	5 (33.3%)	7 (70%)	*p* = 0.07
	No	10 (66.7%)	3 (30%)	
high risk (NA = 2)	Yes	8 (53.3%)	8 (80%)	*p* = 0.17
	No	7 (46.7%)	2 (20%)	
ISS-R (NA = 13)	1	0 (0%)	1 (14.3%)	*p* = 0.23
	2	5 (71.4%)	2 (28.6%)	
	3	2 (28.6%)	4 (57.1%)	
Heavy chains (NA = 0)	BJ	3 (18.8%)	3 (27.3%)	*p* = 0.33
	IgA	1 (6.2%)	3 (27.3%)	
	IgG	11 (68.8%)	5 (45.5%)	
	others	1 (6.2%)	0 (0%)	
Light chains (NA = 0)	Kappa	16 (100%)	4 (36.4%)	*p* = 2e-04
	Lambda	0 (0%)	7 (63.6%)	
Remission status prior to intervention (NA = 0)	CR	3 (18.8%)	0 (0%)	*p* = 0.32
	VGPR	2 (12.5%)	0 (0%)	
	PR	1 (6.2%)	2 (18.2%)	
	MR	0 (0%)	1 (9.1%)	
	SD	1 (6.2%)	1 (9.1%)	
	PD	9 (56.2%)	7 (63.6%)	
Death status (NA = 0)	0	15 (93.8%)	6 (54.5%)	*p* = 0.02
	1	1 (6.2%)	5 (45.5%)	
Age (NA = 0)	Median	65	58	*p* = 0.04
	IQR	3.75	9.5	
Age first diagnosed (NA = 0)	Median	56	51	*p* = 0.11
	IQR	4	6.5	
Number of prior therapy lines(NA = 0)	Median	8.5	7	*p* = 0.23
	IQR	3.25	3	
Days from first diagnosis to intervention(NA = 0)	Median	2797	1168	*p* = 0.001
	IQR	1040.25	863	
Beta-2 microglobulin prior to intervention (NA = 9)	Normal	9 (81.8%)	1 (14.3%)	*p* = 0.01
	High (>3.5 mg/l)	0 (0%)	2 (28.6%)	
	Very high (>5.5 mg/l)	2 (18.2%)	4 (57.1%)	
Albumin prior to intervention (NA = 4)	Low (<35 g/l)	4 (30.8%)	4 (40%)	*p* = 0.64
	Normal	9 (69.2%)	6 (60%)	
LDH prior to intervention (NA = 0)	Normal	5 (31.2%)	3 (27.3%)	*p* = 0.82
	High (>3.75 µkat/l male > 3.55 µkat/l female)	11 (68.8%)	8 (72.7%)	
Creatinine prior to intervention (NA = 0)	Normal	12 (75%)	9 (81.8%)	*p* = 0.68
	High (>114.4 µmol/l male > 96.8 µmol/l female)	4 (25%)	2 (18.2%)	
GFR prior to intervention (NA = 0)	Normal	15 (93.8%)	10 (90.9%)	*p* = 0.78
	High (>114.4 µmol/l male > 96.8 µmol/l female)	1 (6.2%)	1 (9.1%)	
Hemoglobin prior to intervention (NA = 0)	Low (<8.69 mmol/l male < 7.45 mmol/l female)	14 (87.5%)	11 (100%)	*p* = 0.22
	Normal	2 (12.5%)	0 (0%)	
Calcium prior to intervention (NA = 1)	Normal	16 (100%)	9 (90%)	*p* = 0.2
	High (>2.75 mmol/l)	0 (0%)	1 (10%)	
Beta-2 microglobulin post intervention (NA = 6)	Normal	5 (45.5%)	4 (40%)	*p* = 0.21
	High (>3.5 mg/l)	5 (45.5%)	2 (20%)	
	Very high (>5.5 mg/l)	1 (9.1%)	4 (40%)	
Albumin post intervention (NA = 1)	Low (<35 g/l)	3 (20%)	2 (18.2%)	*p* = 0.91
	Normal	12 (80%)	9 (81.8%)	
LDH post intervention (NA = 1)	Normal	5 (31.2%)	3 (30%)	*p* = 0.95
	High (>3.75 µkat/l male > 3.55 µkat/l female)	11 (68.8%)	7 (70%)	
Creatinine post intervention (NA = 0)	Normal	11 (68.8%)	7 (63.6%)	*p* = 0.78
	High (>114.4 µmol/l male > 96.8 µmol/l female)	5 (31.2%)	4 (36.4%)	
GFR post intervention (NA = 0)	Low (<40 ml/min)	2 (12.5%)	2 (18.2%)	*p* = 0.68
	Normal	14 (87.5%)	9 (81.8%)	
Hemoglobin post intervention (NA = 1)	Low (<8.69 mmol/l male < 7.45 mmol/l female)	14 (93.3%)	11 (100%)	*p* = 0.38
	Normal	1 (6.7%)	0 (0%)	
CRS status post intervention (NA = 0)	No CRS	2 (12.5%)	3 (27.3%)	*p* = 0.55
	CRS without Tocilizumab	9 (56.3%)	6 (54.5%)	
	CRS with Tocilizumab	5 (31.2%)	2 (18.2%)	

Overview of the characteristics of 27 RRMM patients treated with Ide-cel. Grouping was performed according to response after CAR T cell treatment into non-PD and PD patients. CRS post CAR T cell treatment was evaluated by applying published guidelines [26]. Chi-Square test was used to calculate *p*-values for nominal and ordinal features. In case of continuous features *p*-values were calculated with the Mann–Whitney U test.

### Expansion of CAR T cells is associated with superior outcome

**Fig. 2:**
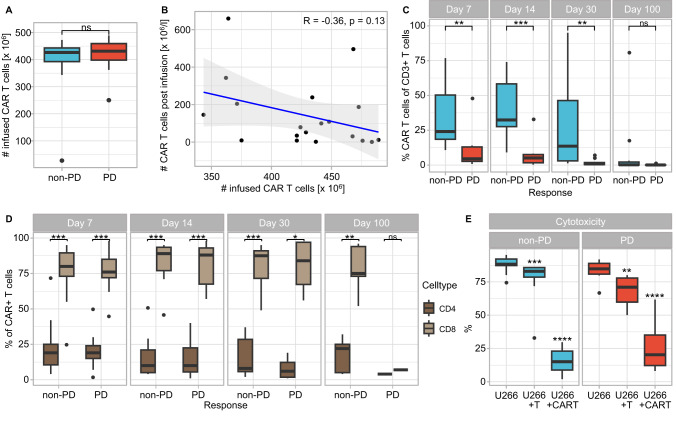
Infused CAR T cells, characteristics of expanded CAR T cells as well as CAR T cell functionality. **A** Absolute number of infused CAR T cells classified according to their later response into patients without (non-PD) and with progressive disease (PD). **B** Correlation of infused CAR T cell numbers and expanded CAR T cells detected 14 days after infusion in PB. Mapped are individual values (dots) as well as Pearson correlation (blue line with gray confidence interval). **C** Proportion of CAR T cells within CD3^+^ T cells as well as **D** distribution of CD4^+^ and CD8^+^ cells within CD3^+^ CAR T cells in PB of non-PD and PD patients 7, 14, 30, and 100 days after CAR T cell infusion. **E** Assessment of in vitro toxicity of T and CAR T cells on the myeloma cell line (U266). Depicted are the percentages of living U266 cells 24 h after cultivation without additional cells (U266) and co-cultured with T cells isolated from PB of non-PD and PD patients on day 0 (U266+T) as well as on day 7 after CAR T cell infusion (U266+CAR T). Mann–Whitney *U* test was used to calculate significances between **A**, **C** non-PD and PD patients, **D** CD4 and CD8 CAR T cells as well as **E** U266 cells without and with the addition of effector cells (either T or CAR T cells). *p < 0.05, **p < 0.01, ***p < 0.001, ****p < 0.0001, ns not significant.

To assess CAR T cell dynamics within PB, we first checked the amount of infused CAR T cells. The median number of infused CAR T cells was 430 × 10^6^ (range: 248.8 – 489.1 × 10^6^) with one exception (25.7 × 10^6^, out of specification product). However, the quantity showed no correlation to response (Fig. 2A) or the capacity for in vivo expansion of CAR T cells post-infusion (Fig. 2B).

Next, we quantified expansion capacity and observed significantly reduced numbers of CD3^+^ CAR T cells on days 7 (*p* < 0.01), 14 (*p* < 0.001), and 30 (*p* < 0.01) in PD as compared to non-PD patients (Fig. 2C).

A more detailed characterization of the CD3^+^ CAR T cell compartment demonstrated that it was predominantly comprised of CD8^+^ T cells (Fig. 2D). However, this effect was not related to response, since the longitudinal distribution of CD4^+^ and CD8^+^ CAR T cells was similar in patients with and without PD.

### CAR T cells of responders and non-responders show comparable in vitro activity

In order to check functionality of CAR T cells in vitro, we isolated PBMCs at days 0 and 7. After CD3/CD28 stimulation, expanded (CAR) T cells were co-cultured with a BCMA-expressing MM cell line (U266). Whereas incubation for 24 h with T cells isolated on day 0 resulted only in a slight reduction of U266 cells, significantly reduced frequencies of U266 cells co-cultured with cells isolated on day 7 were observed (*p* < 0.0001, Fig. 2E). This effect was similar in both patient groups (PD and non-PD), illustrating that functionality of CAR T cells of non-responders was not restricted.

### Differences between responders and non-responders at time of leukapheresis and after LDP

**Fig. 3:**
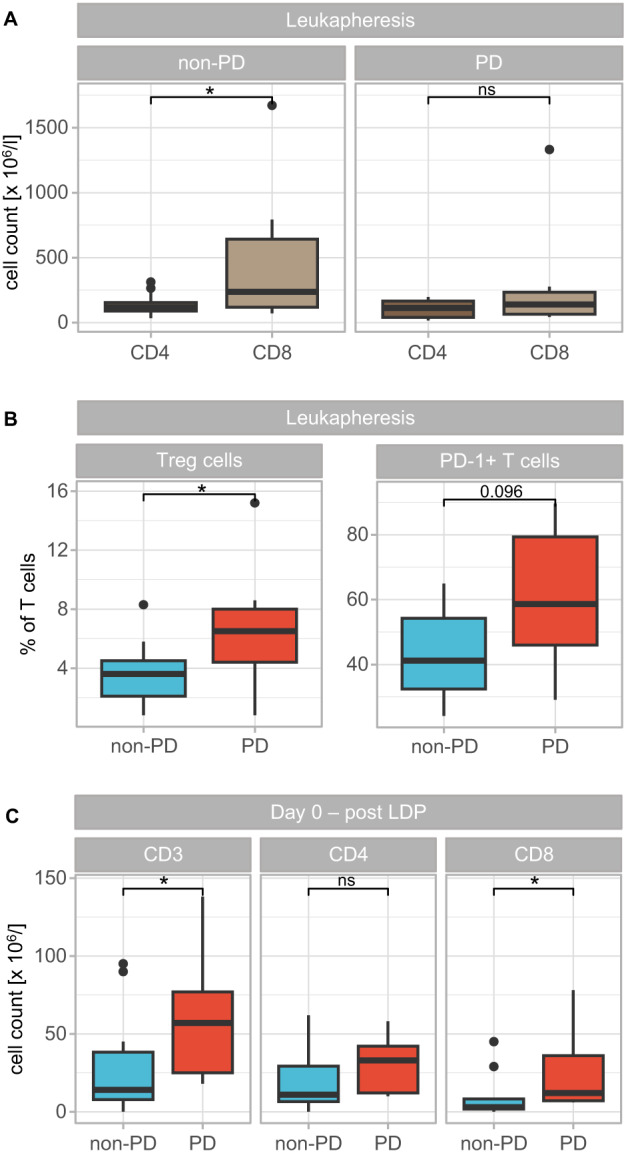
Differences in T cell compartment of non-PD and PD patients at time of leukapheresis and after LDP before CAR T cell infusion. **A** Absolute cell counts of CD3^+^CD4^+^ and CD3^+^CD8^+^ T cells in PB of non-PD and PD patients at day of leukapheresis. **B** Proportion of CD25^+^CD127^low^ Treg cells (left) and PD-1^+^ T cells (right) within CD3^+^ T cell population in PB of non-PD and PD patients at day of leukapheresis. **C** Absolute numbers of CD3^+^, CD3^+^CD4^+^, and CD3^+^CD8^+^ T cells in PB of non-PD and PD patients after LDP at day 0 (before CAR T cell infusion). Significances were calculated with the Mann–Whitney U test between **A** CD4^+^ and CD8^+^ T cells as well as **B**, **C** non-PD and PD patients. *p < 0.05, ns not significant.

To estimate the composition of the infusion product, we assessed the starting point for CAR T cell production by checking the entire T cell compartment in PB at time of leukapheresis. Remarkably, already at this time point significantly more CD8^+^ T cells as compared to CD4^+^ T cells were observed in responders (*p* < 0.05, Fig. 3A). In contrast, numbers of CD4^+^ and CD8^+^ T cells were comparable in PD patients.

Further analysis of the T cells revealed that a significantly increased proportion belonged to Treg cells within PB of PD as compared to non-PD patients at day of leukapheresis (*p* < 0.05, Fig. 3B, left). In addition, there was a trend towards higher expression levels of the activation and exhaustion marker PD-1 on CD3^+^ T cells from PD patients (*p* = 0.096, Fig. 3B, right).

Furthermore, we analyzed the effectiveness of LDP, which strongly reduces the number of peripheral T cells. In non-responders, LDP seemed to be not as effective as in responding patients, since significantly more CD3^+^ T cells and in particular CD3^+^CD8^+^ T cells were still detectable on day 0 (p < 0.05, Fig. 3C).

These results illustrate that success of Ide-cel therapy is already affected by the T cell composition of PB at time of leukapheresis as well as by the success of LDP prior to CAR T cell infusion.

### Successful CAR T cell therapy impacts bystander T cells

**Fig. 4:**
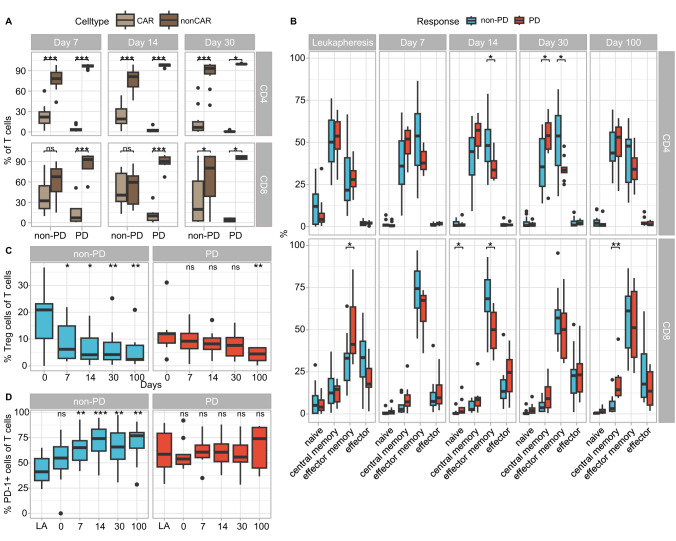
Effects of CAR T cell infusion of bystander T cells. **A** Distribution of CAR and non-CAR CD4^+^ (top) as well as CD8^+^ (bottom) T cells in PB of non-PD and PD patients 7, 14, and 30 days after CAR T cell infusion. **B** Proportions of naïve (CD45RA^+^CD45RO^-^CCR7^+^), central memory (CD45RA^-^CD45RO^+^CCR7^+^), effector memory (CD45RA^-^CD45RO^+^CCR7^-^), and effector (CD45RA^+^CD45RO^-^CCR7^-^) CD4^+^ (top) as well as CD8^+^ (bottom) T cells in PB of non-PD and PD patients at day of leukapheresis and 7, 14, 30, and 100 days after CAR T cell infusion. **C** Percentages of CD25^+^CD127^low^ Treg cells as well as **D** PD-1^+^ cells within CD3^+^ T cell compartment in PB of non-PD and PD patients on day of leukapheresis (**D**) and on days 0, 7, 14, 30, and 100 (**C**, **D**). Mann–Whitney *U* test was performed to calculate significances between **A** CAR and non-CAR T cells, **B** non-PD and PD patients, **C** day 0 and days after CAR T cell infusion (7, 14, 30, and 100) and **D** day of leukapheresis (LA) and days 0, 7, 14, 30 and 100. *p < 0.05, **p < 0.01, ***p < 0.001, ns not significant.

In order to determine the effect of CAR T cells on bystander cells, we analyzed differences in the entire T cell compartment post-infusion. At all analyzed time points a mixture of CAR and non-CAR T cells was present (Fig. 4A). For CD4^+^ T cells the vast majority were untransfected at all time points and regardless of response. However, the distribution of CAR and non-CAR CD8^+^ T cells differed between PD and non-PD patients. In responders, the percentages of CD8^+^ CAR T cells approximated CD8^+^ non-CAR T cells 7 and 14 days after CAR T cell infusion. In contrast, CD8^+^ non-CAR T cells prevailed at all time points in non-responders, presumably due to the increased number of CD8^+^ T cells prior to infusion, which reduced the capability of CAR T cell expansion.

Analysis of the T cell differentiation (Fig. 4B) showed that while PBMCs at day of leukapheresis still contained around 10–15% naïve CD4^+^ T cells regardless of later response, after CAR T cell infusion naïve CD4^+^ T cells were negligible and almost the entire population exhibited either a central memory or an effector memory phenotype.

Regarding CD8^+^ T cell differentiation shortly after CAR T cell infusion, effector memory CD8^+^ T cells outbalanced all other cell types. This effect was even more pronounced in non-PD patients, especially at early time points. Additionally, the proportion of effector CD8^+^ T cells increased over time.

Further analysis of the T cell compartment revealed a progressively reducing proportion of Treg cells after CAR T cell infusion in PD and non-PD patients (Fig. 4C). Simultaneously, PD-1-expressing T cells increased significantly post-infusion as compared to time of leukapheresis in responding patients (*p* < 0.01, Fig. 4D). In contrast, in PD patients the proportion of T cells expressing PD-1 remained on a constantly high level similar to the time of leukapheresis.

### CRS and CAR T cell dynamics

After CAR T cell infusion, most patients experienced a CRS grade I or II and required tocilizumab application (no CRS: *n* = 5, CRS/without tocilizumab: *n* = 15, CRS/with tocilizumab: *n* = 7). However, the occurrence of CRS and the treatment with tocilizumab did not affect the outcome of Ide-cel treatment (Table 1). No cases of neurotoxicity were observed.

**Fig. 5:**
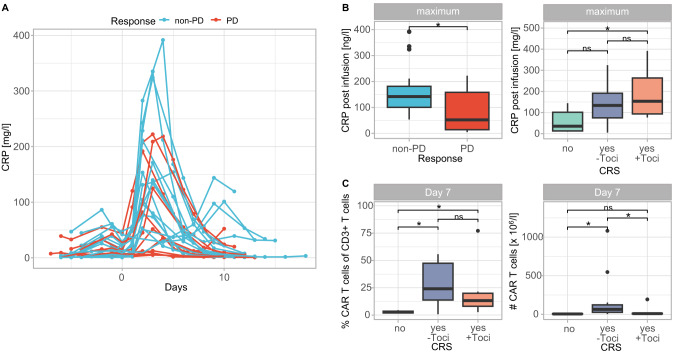
Dynamics of CRP levels during CAR T cell therapy and CAR T cell expansion with regard to CRS. **A** Serum CRP levels during the in-patient stay for CAR T cell infusion. Depicted is the course for every individual patient color-coded by response (non-PD: blue, PD: red). **B** Comparison of the maximum CRP level in serum of patients classified by response (left) and CRS occurrence (right). **C** Proportions of CAR T cells within CD3^+^ T cell compartment (left) as well as absolute numbers of CAR T cells (right) in PB of patients classified by CRS occurrence, 7 days after CAR T cell infusion. Significances were calculated using the Mann–Whitney U test. *p < 0.05, ns not significant.

Analysis of serum C-reactive protein (CRP) levels revealed a strong increase shortly after infusion in most patients without evidence for microbial infections (Fig. 5A). Comparison of the maximum CRP value during the in-patient stay (Fig. 5B) showed a significant increase in patients with response as compared to PD patients (*p* < 0.05), reflecting a connection between a cytokine-driven inflammation and response. The maximum CRP level was elevated in patients with CRS as compared to patients without CRS and was highest in patients with CRS and tocilizumab treatment (*p* < 0.05). In fact, 30 days after CAR T cell treatment CRP values normalized in all patients.

Associating CAR T cell expansion with CRS showed significant differences shortly after CAR T cell infusion (Fig. 5C, D). Whereas percentages of CD3^+^ CAR T cells were significantly elevated in all patients experiencing CRS (*p* < 0.05) shortly after infusion (Fig. 5C), absolute numbers were significantly increased only in patients without tocilizumab (*p* < 0.05, Fig. 5D), illustrating that CD3^+^ T cells were diminished by tocilizumab. This indicates that CAR T cell expansion is accompanied by an immune cell activation reflected by symptoms of CRS.

### CRS and prolonged cytopenia

**Fig. 6:**
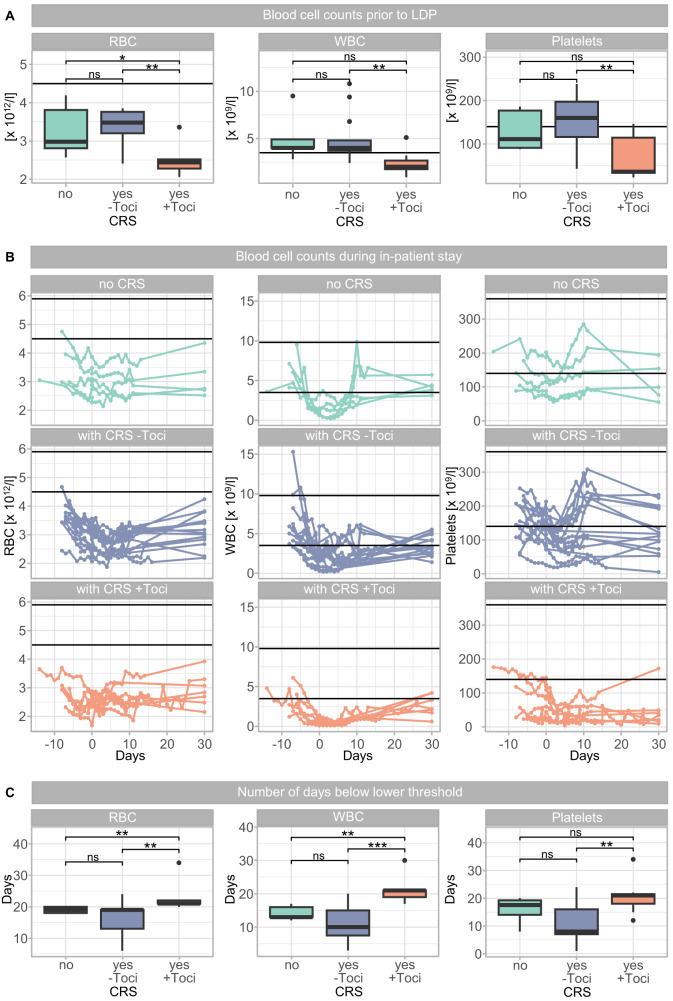
Blood cell counts during CAR T cell therapy. **A** Cell counts of red blood cells (RBC, left), white blood cells (WBC, middle), and platelets (right) in PB of patients classified according to later CRS occurrence (after CAR T cell infusion) prior to LDP. Horizontal lines indicate lower thresholds for each cell line (RBC: 4.5 × 10^12^/l, WBC: 3.5 × 10^9^/l, platelets: 140 × 10^9^/l). **B** Course of RBC (left), WBC (middle), and platelets (right) in every individual patient classified by CRS occurrence into patients without CRS (no CRS, top row), with CRS, but without tocilizumab requirement (with CRS –Toci, middle row) and with CRS and with tocilizumab treatment (with CRS +Toci, bottom row). Horizontal lines indicate threshold ranges for each cell line (RBC: 4.5–5.9 × 10^12^/l, WBC: 3.5–9.8 × 10^9^/l, platelets: 140–360 × 10^9^/l). **C** Days below the lower threshold during the in-patient stay of patients without CRS, with CRS but without tocilizumab requirement as well as with CRS and with tocilizumab treatment. For the calculation of significances Mann–Whitney *U* test was used. *p < 0.05, **p < 0.01, ***p < 0.001, ns not significant.

Analysis of the complete blood counts prior to LDP revealed that patients experiencing CRS with tocilizumab requirement had already significantly less red and white blood cells as well as platelets (*p* < 0.01, Fig. 6A).

Furthermore, during the in-patient stay, the trend for all three blood cell lines was consistent for the entire cohort, with an initial drop caused by LDP and a steady recovery (Fig. 6B). By day 30 most patients showed a blood cell count similar to the ones detected before LDP. However, classification of the patients according to CRS status showed, that while patients without CRS and with CRS but without tocilizumab treatment were almost undistinguishable from each other, patients receiving tocilizumab experienced more severe and longer persisting cytopenia in all three cell lines. This was reflected by a significantly increased number of days below lower limit (*p* < 0.01 for red and white blood cells, respectively, Fig. 6C).

Collectively, the analysis of PB cells illustrated that most patients suffered from pancytopenia, with the strongest manifestation in patients with CRS and tocilizumab treatment.

## Discussion

FDA and EMA approval of Ide-cel revolutionized the treatment landscape for RRMM. However, since BCMA-CAR T cell dynamics as well as the effects on bystander cells and side effects are still incompletely understood, we addressed these aspects in the current study. We identified pre-infusional characteristics – including elevated sBCMA levels and insufficient LDP – that were associated with adverse outcome and showed that CAR T cell expansion is correlated to superior response and the occurrence of CRS. The latter was accompanied by prolonged cytopenia. While in vitro activity of extracted CAR T cells was comparable between responders and non-responders, significant effects on bystander T cells were observed.

For CD19-directed CAR T cells, it has been shown that CAR T cell kinetics comply with three different phases - expansion, contraction, and persistence. Response and outcome are correlated to CD19-directed CAR T cell expansion in patients with B-cell neoplasia [1, 4–8]. In line with the real-world analysis by Sanoyan et al. [13], our flow cytometry-based analysis revealed similar phases in BCMA-CAR T cell-treated RRMM patients, with a peak expansion around two weeks after infusion and a rapid decline afterwards. In addition, the lack of expansion was accompanied by an absent response and early progression of the disease.

Our analyses showed that the amount of infused CAR T cells did not correlate with the maximal in vivo expansion or response, as seen in the CD19-CAR T cell product Tisagenlecleucel (Tisa-cel) [1, 14].

In contrast to CD19-targeting CAR T cells in acute lymphoblastic leukemia (ALL) and diffuse large B-cell lymphoma (DLBCL), which require long-term persistence for durable remissions [1, 2], we observed that CAR T cells were undetectable in PB 100 days after infusion in most patients, even in those with sustained response. This additionally contrasts the KarMMa trial, in which CAR T cell levels persisted for up to 12 months [10]. These differences could be explained by varying analysis methods. While quantitative polymerase chain reaction (qPCR) quantifies CAR transgene levels with higher sensitivity enabling the tracking of CAR T cells over a longer period, flow cytometry directly detects CAR-expressing T cells [14]. Even though both approaches showed a strong correlation, some patients with expanded CAR transgene levels had missing protein levels, indicating the lack of functional expansion [15]. Thus, flow cytometry might be a better predictor for CAR T cell activity.

Our post-infusion analysis revealed that CD8^+^ CAR T cells dominated the compartment, aligning with a study of a non-commercial BCMA-CAR T cell [8]. It has been demonstrated that this shift towards CD8^+^ CAR T cells in responders was already present in some CD19-targeting infusion product [16]. Furthermore, Li et al. indicated limited relevance of CD4^+^ CAR T cells due to strongly reduced cell counts post-infusion as well as less cytotoxic activity [17]. Our data show that similar to Tisa-cel-treated patients [18], after CAR T cell infusion, naïve CD8^+^ T cells (CD45RA^+^CD45RO^-^CCR7^+^) are almost absent and CD8^+^ T cells are predominantly characterized by an effector memory phenotype (CD45RA^-^CD45RO^+^CCR7^-^), transitioning to effector subtype (CD45RA^+^CD45RO^-^CCR7^-^). Absence of naïve and central memory T cells post-infusion explains the short PB persistence of CAR T cells, since effector memory T cells exhibit low self-renewal and survival capacity [16, 19]. Additionally, in responders, T cells expressing the activation and exhaustion marker PD-1 significantly increased over time, which is in line with the study from Brudno et al. showing a more differentiated phenotype as well as higher fraction of cells expressing senescent markers and reduced T cell proliferative capacity after CAR T cell infusion [8]. However, exhausted CAR T cells are not inert, since they still have a killing ability in vitro equally true for our setting and CD19-directed CAR T cells [16]. Thus, we hypothesize that lack of response is caused by the diminished in vivo expansion rather than the functionality of CAR T cells.

Guarini et al. demonstrated that CD19-directed CAR T cell treatment of DLBCL and ALL patients induces a reshaping of the immune system by increasing T cell counts and inducing cytokine production [20]. Since, after Ide-cel infusion we mainly detected CAR-negative CD4^+^ T cells, this seemed to be also relevant for BCMA-directed CAR T cells.

Sources for non-CAR T cells after LDP could be either residual T cells, not depleted by the respective regime or untransfected T cells within the infusion product. Non-CAR T cells have been shown to produce granzymes and cytokines with anti-tumor activity [3]. Thus it is presumed that CAR T cells exert their anti-tumor immunity by two different strategies: direct killing via the infused CAR T cells and activation of the local immune response.

Furthermore, the composition of CD4^+^ T cells is altered post-infusion, with a progressively reducing proportion of Treg cells over time, which has been equally described for CD19-CAR T cells [20]. The presence of Treg cells in tumors inhibit the anti-tumor response and hence induces disease progression [19]. Since, we could not observe a difference of Treg cell frequencies after infusion with regard to response, we can conclude that Treg cells were not decisive for the outcome in our setting.

Our data show, that cellular composition at day of leukapheresis already determine response to Ide-cel, with elevated CD8^+^ T cell proportions improving the therapeutic success. Conversely, a study by Garfall et al. comparing leukapheresis products from early-stage MM and heavily pretreated RRMM patients showed a higher CD4/CD8 ratio in the early-stage cohort [21]. Supported by their previous study on BCMA-directed CAR T cells [22], they concluded that leukapheresis products from the early-stage cohort might be more effective than from heavily pretreated patients. Differences in CD4/CD8 T cell distribution in our model may result from varying in vitro BCMA-CAR T cell generation procedures. Additionally, their preclinical validation showed greater anti-tumor activity in CD8^+^ T cells [23] and treated RRMM patients had predominantly activated CD8^+^ CAR T cells [22], underlining the relevance of CD8+ T cells.

Furthermore, non-responders had significantly more Treg cells in PB at day of leukapheresis, mirroring findings from CD19-directed CAR T cells, where CAR-transduced Treg cells reduced treatment efficacy [18]. However, since Treg cell proportions did not differ post-infusion with regard to response suggests that Ide-cel production may aim to minimize Treg cell-related issues.

In addition, responders had fewer PD-1-expressing CD3^+^ T cells and significantly reduced proportion of CD8^+^ T cells exhibiting an effector memory phenotype (CD45RA^-^CD45RO^+^CCR7^-^) at time of leukapheresis. Similar finding were reported for BCMA-CAR T cells [21, 22] and CD19-CAR T cells [16, 19], showing that CAR T cells produced from less differentiated T cells possess greater anti-tumor activity and proliferation potential. This illustrates that a more differentiated as well as exhausted T cell phenotype at day of leukapheresis is accompanied with a worse outcome after CAR T cell treatment. Additionally, we hypothesize that an insufficient LDP results in a suboptimal response, supported by Cohen et al. demonstrating improved CAR T cell expansion in patients receiving cyclophosphamide conditioning [22].

CAR T cell therapy is often accompanied by CRS and neurotoxicity symptoms, ranging from mild symptoms to life-threatening reactions. However, it has been shown, that higher-grade CRS and neurotoxicity was less frequent in RRMM patients [24]. In contrast to CD19, which is expressed by a large variety of hematopoietic cells, BCMA is mainly expressed on plasmablasts and plasma cells. Thus, on-target/off-tumor toxicity is supposed to be reduced in BCMA-directed CAR T cells.

Our findings, along with the real-world study by Sanoyan et al. [13] support these observations. Similar to the KarMMa trial [10], 81% of our patients developed CRS and 32% of these patients required tocilizumab treatment. Hematologic toxicity, characterized by anemia, neutropenia, and thrombocytopenia, was observed in all patients, which aligns with the findings of Sanoyan et al. [13]. Additionally, a subgroup of patients had significantly reduced cell counts for all three lines even before LDP, and interestingly, all of these patients developed CRS requiring tocilizumab treatment after CAR T cell infusion.

The group of Subklewe et al. developed a CAR-HEMATOTOX score to predict hematotoxicity after CAR T cell treatment [25]. In RRMM patients a high CAR-HEMATOTOX score prior to LDP is a predictor for severe toxicity events after CAR T cell infusion [24]. Additionally, a high score is also a predictor for inferior response and survival outcome in RRMM. While our results only partially confirm these predictions, as we did not observe any correlation between CRS and response, it is worth noting that our analyses focus solely on cytopenia and did not include baseline CRP or ferritin values used in CAR-HEMATOTOX score calculation. Furthermore, we found that the tocilizumab administration in higher-grade CRS patients did not interfere with response, similar as described for Tisa-cel-treated patients [15].

Our results revealed a correlation between CRS and CAR T cell expansion shortly after infusion, highlighting that increased expansion is accompanied by enhanced immune cell activation, as reflected by elevated CRP values. CD19-directed CAR T cells showed diverse results: while Axicabtagene ciloleucel (Axi-cel)-treatment did only show an association between peak expansion and neurological events but not CRS [5], a positive correlation of high-grade CRS with elevated CAR T cell expansion was reported for Tisa-cel [15].

Major downside of our study is the relatively small number of analyzed patients. Larger cohorts with detailed analyses, e.g. from flow cytometry are currently only available from clinical trial populations. With the growing number of patients being treated with CAR T cells outside clinical trials, further studies will follow to confirm and expand our current hypotheses.

Taken together, we demonstrated that initial CAR T cell expansion, predominantly caused by effector memory CD8^+^ CAR T cells, was linked to response and CRS in Ide-cel-treated RRMM patients and that the persistence of CAR T cells in PB was not essential for durable remission. Furthermore, our data provide first evidence that responders and non-responders can early be distinguished by differential cellular composition in CAR and non-CAR T cell compartments with distinct features already present at day of leukapheresis. The data showed that a containment of the disease burden prior to CAR T cell therapy is one of the most important features for the success of the therapy. Therefore, future studies are needed to determine the optimal use and modality of bridging therapies.

### Supplementary information

Supplemental Figure 1

Supplemental Figure 2

## Data Availability

For original data, please contact Maximilian.merz@medizin.uni-leipzig.de.
