# Supplementary material for: Cellular dynamics following CAR T cell therapy are associated with response and toxicity in relapsed/refractory myeloma
Source: Leukemia. 2024 Jan 6;38(2):372–82. doi: 10.1038/s41375-023-02129-y (PMC10844085; doi:10.1038/s41375-023-02129-y)
Supplement: Supplementary file 1 — Supplemental Figure 1 [file 41375_2023_2129_MOESM1_ESM.pdf]

Supplemental figure 1

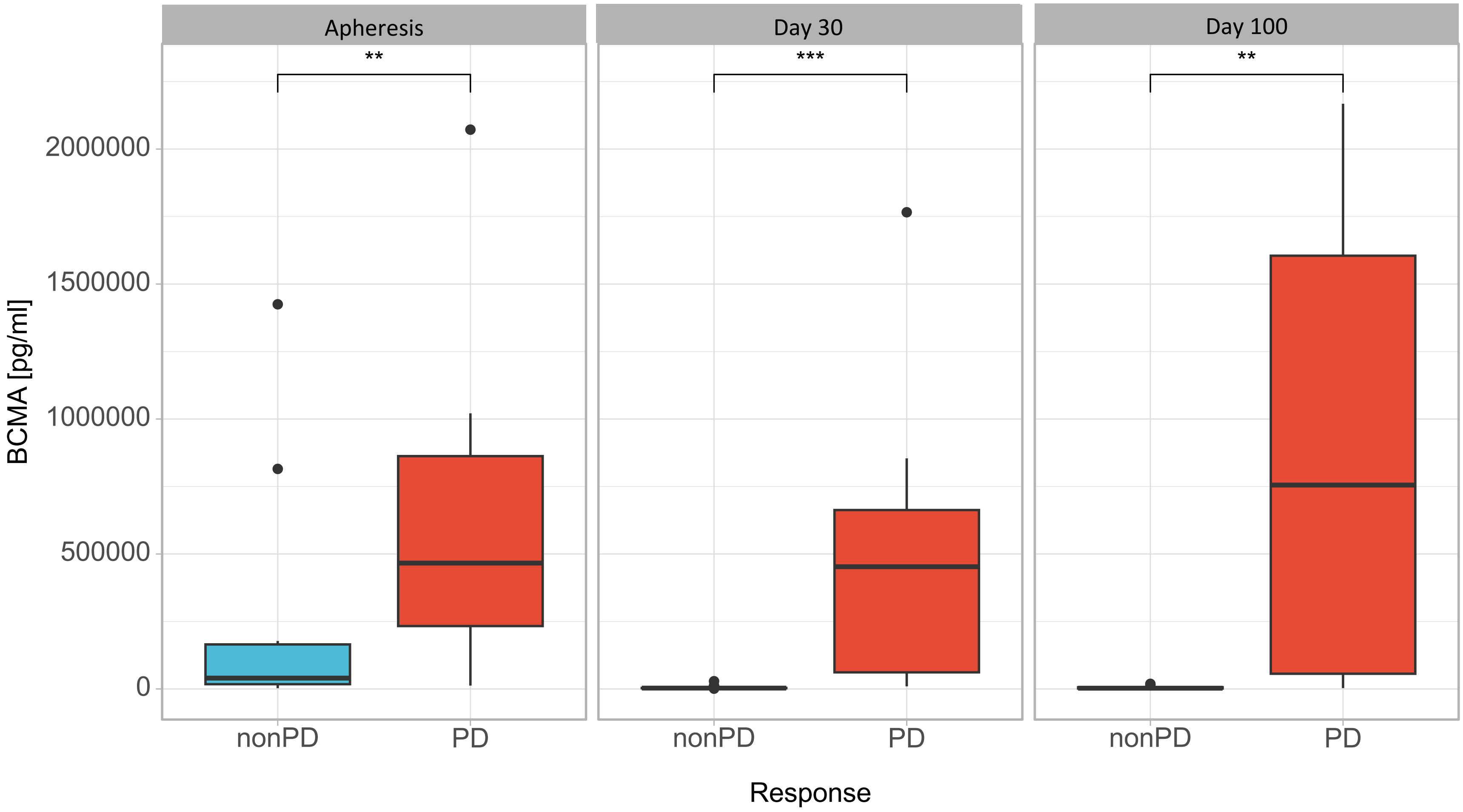

Supplemental Figure 1: Comparison of sBCMA levels between nonPD and PD patients

Soluble BCMA (sBCMA in pg/ml on x-axis) levels in serum were measured at time of leukapheresis (apheresis) and days 30 and 100 following CAR T cell infusion (y-axis). Significant differences between both groups were assessed at all three different timepoints. For the calculation of significances Mann-Whitney U test was used. ** $p < 0.01$, *** $p < 0.001$.
