# Supplementary material for: Cellular dynamics following CAR T cell therapy are associated with response and toxicity in relapsed/refractory myeloma
Source: Leukemia. 2024 Jan 6;38(2):372–82. doi: 10.1038/s41375-023-02129-y (PMC10844085; doi:10.1038/s41375-023-02129-y)
Supplement: Supplementary file 2 — Supplemental Figure 2 [file 41375_2023_2129_MOESM2_ESM.pdf]

Supplemental figure 2

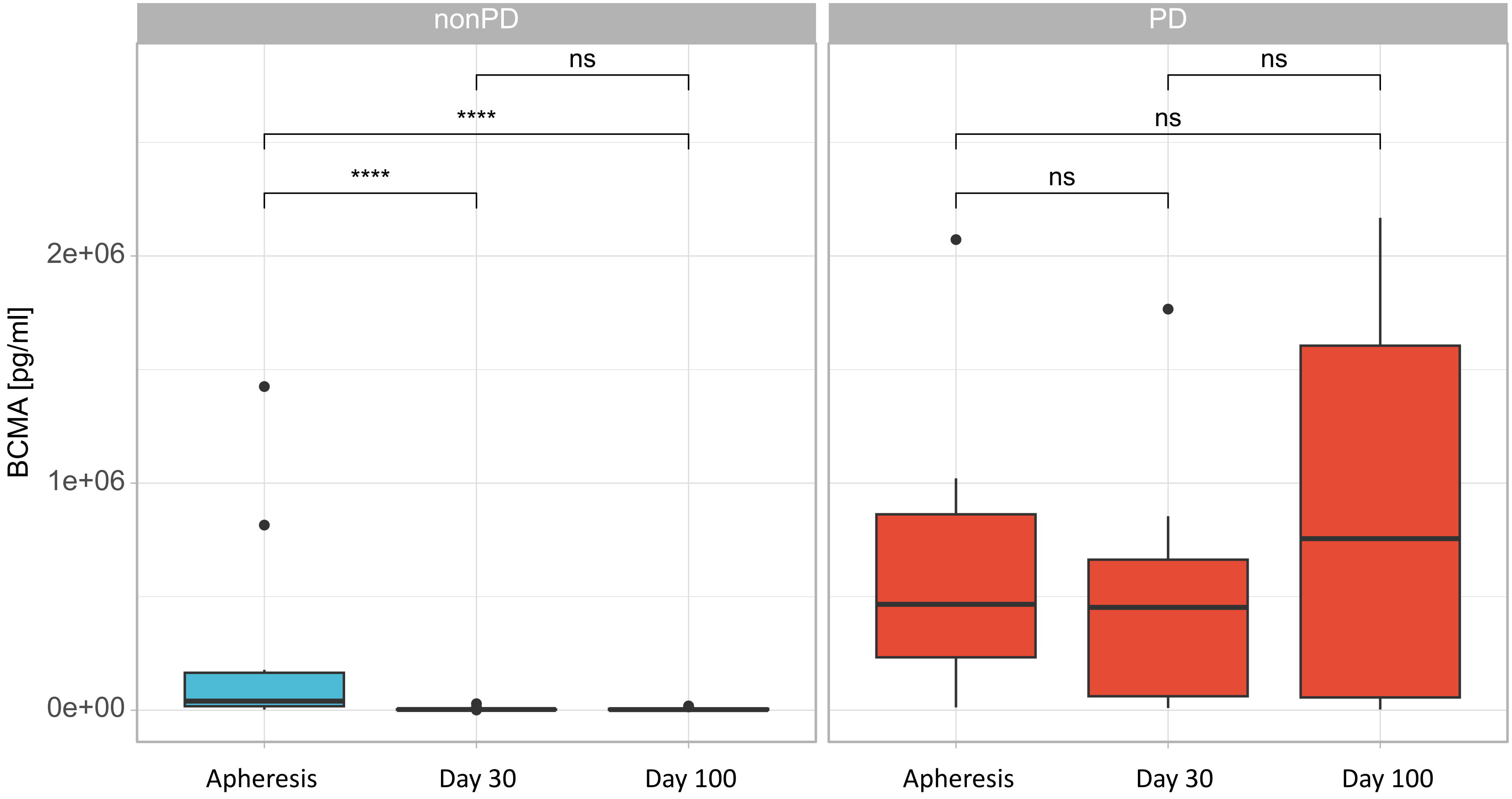

Supplemental Figure 2: Longitudinal comparison of sBCMA levels in nonPD and PD patients
Soluble BCMA (sBCMA in pg/ml on x-axis) levels in serum were measured at time of leukapheresis (apheresis) and days 30 and 100 following CAR T cell infusion (y-axis). In nonPD patients, sBCMA levels decreased significantly compared to baseline samples (apheresis) following CAR T cell infusion. No significant differences between all three timepoints were assessed in PD patients, reflecting no decrease in tumor burden. No loss of BCMA expression was observed in both cohorts. For the calculation of significances Mann-Whitney U test was used. **** $p < 0.0001$, ns = not significant.
